# Mapping Zika virus infection using geographical information systems in Tolima, Colombia, 2015-2016

**DOI:** 10.12688/f1000research.8436.1

**Published:** 2016-04-05

**Authors:** Alfonso J. Rodriguez-Morales, Maria Leonor Galindo-Marquez, Carlos Julian García-Loaiza, Juan Alejandro Sabogal-Roman, Santiago Marin-Loaiza, Andrés Felipe Ayala, Carlos O. Lozada-Riascos, Andrea Sarmiento-Ospina, Heriberto Vásquez-Serna, Carlos E. Jimenez-Canizales, Juan Pablo Escalera-Antezana

**Affiliations:** 1 Public Health and Infection Research Group, Universidad Tecnologica de Pereira, Pereira, Colombia; 2 Organización Latinoamericana para el Fomento de la Investigación en Salud (OLFIS), Riohacha, Colombia; 3 Colombian Collaborative Network of Zika (RECOLZIKA), Pereira, Colombia; 4 Regional Information System, Universidad Tecnológica de Pereira, Pereira, Colombia; 5 Secretary of Health of Ibagué, Ibagué, Colombia; 6 Secretary of Health of Tolima, Ibagué, Colombia; 7 Tongji Hospital - Tongji Medical College, Huazhong University of Science and Technology, Wuhan, China

**Keywords:** Zika, epidemiology, public health, travelers, Colombia, Latin America.

## Abstract

*Objective:* Geographical information systems (GIS) have been extensively used for the development of epidemiological maps of tropical diseases, however not yet specifically for Zika virus (ZIKV) infection.

*Methods: *Surveillance case data of the ongoing epidemics of ZIKV in the Tolima department, Colombia (2015-2016) were used to estimate cumulative incidence rates (cases/100,000 pop.) to develop the first maps in the department and its municipalities, including detail for the capital, Ibagué. The GIS software used was Kosmo Desktop 3.0RC1®. Two thematic maps were developed according to municipality and communes incidence rates.

*Results: *Up to March 5, 2016, 4,094 cases of ZIKV were reported in Tolima, for cumulated rates of 289.9 cases/100,000 pop. (7.95% of the country). Burden of ZIKV infection has been concentrated in its east area, where municipalities have reported >500 cases/100,000 pop. These municipalities are bordered by two other departments, Cundinamarca (3,778 cases) and Huila (5,338 cases), which also have high incidences of ZIKV infection. Seven municipalities of Tolima ranged from 250-499.99 cases/100,000 pop., of this group five border with high incidence municipalities (>250), including the capital, where almost half of the reported cases of ZIKV in Tolima are concentrated.

*Conclusions:* Use of GIS-based epidemiological maps helps to  guide decisions for the prevention and control of diseases that represent significant issues in the region and the country, but also in emerging conditions such as ZIKV.

## Introduction

Zika virus (ZIKV) epidemics are progressing across most of the territories of Latin America without effective control ^1^. In particular, some areas of Colombia are being impacted with a high incidence of cases, nevertheless without show their incidence rates and detailed geographical distribution in most reports. Areas where cocirculation of dengue and chikungunya have occurred ^2, 3^, are particularly at risk. In this setting updated epidemiological information is of utmost importance, which should include the availability of risk maps in order to address recommendations to prioritize interventions as well for the identification of areas of risk by visitors or people returning from visiting specific places ^4, 5^. Accordingly, we have developed epidemiological maps for ZIKV in Colombia using geographical information systems (GIS) at one of the high incidence departments (Tolima) located in the central area of the country. We have previously provided GIS-based epidemiological maps for CHIKV in other areas of the country ^5^.

## Methods

Scientific publications using GIS for development of epidemiological maps in ZIKV lack in Latin America and Colombia. Tolima, a department surrounded by seven departments (five at the west and two at the east) with 47 municipalities (for a total population of 1,412,230 habitants) is one of the territories significantly affected by the 2015–2016 outbreak. Its capital, the Ibagué municipality, constitutes 13 urban communes and a rural area, comprising 39.6% of the total population of the department.

Surveillance case data (2015–2016; officially reported by the National Institute of Health, Colombia) ^6^ were used to estimate the cumulative incidence rates using reference population data (2016), on ZIKV infections (cases/100,000 pop.) and to develop the first maps in the municipalities of Tolima and in the communes of the Ibagué municipality. Data for this study were gathered from 47 primary notification units, one per municipality, and later consolidated at the department level. In the case of the Ibagué municipality, data were collected from healthcare institutions of the 13 communes, and later consolidated at the municipality level. Diagnosis of ZIKV infection included either laboratory and/or syndromic surveillance (clinical definition of fever, rash, conjunctivitis and arthralgias in a municipality with previously ZIKV circulation, at least one case confirmed by RT-PCR). The software Microsoft Access (version 365)® was used to design the spatial database, and to import incidence rates for municipalities in Tolima and communes in Ibagué to the GIS software. The open source GIS software used was Kosmo Desktop 3.0 RC1®. Geographic data (municipalities and department polygons) required for the department and the Ibagué municipality were provided by the Regional Information System of the Coffee-Triangle region. The shapefiles (based on official cartography) of municipalities and communes (.shp) were linked to the data table database through a spatial join operation, in order to produce digital maps of the incidence rates.

## Results

Raw data for 'Mapping Zika virus infection using geographical information systems in Tolima, Colombia, 2015–2016' Click here for additional data file. Copyright: © 2016 Rodriguez-Morales AJ et al. 2016 Data associated with the article are available under the terms of the Creative Commons Zero "No rights reserved" data waiver (CC0 1.0 Public domain dedication).

**Figure 1.**
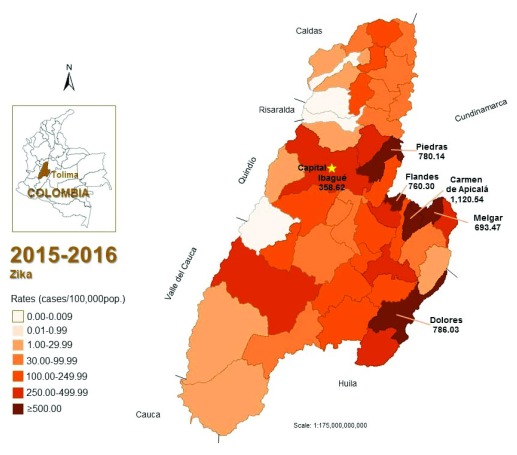
Geographic distribution of ZIKV incidence rates (cases/100,000 pop.) in the Tolima department, Colombia, 2015–2016. (*Up to the 9th epidemiological week, March 5, 2016).

Up to March 5, 2016, 4,094 cases of ZIKV were reported in Tolima (5.93% diagnosed by RT-PCR for ZIKV), for cumulative rates of 289.9 cases/100,000 pop. (7.95% of the country). Rates ranged from 0 to 1,120.5 cases/100,000 pop. (Carmen de Apicalá, 2.4% of the department cases), followed by Dolores (786.0 cases/100,000 pop.; 1.5%), Piedras (780.1 cases/100,000 pop.; 1.1%), Flandes (760.3 cases/100,000 pop.; 5.4%), Melgar (693.5 cases/100,000 pop.; 6.2%) ( Figure 1). These five municipalities (out of 47), reported 16.61% of cases of the department ( Table 1). The capital municipality, Ibagué have reported 2,004 cases (358.6 cases/100,000 pop.; 48.9%) ( Figure 1). The other five municipalities reported incidence rates between 387.3 and 469.2 cases/100,000 pop. These ten territories together with the capital reported more than 83% of the ZIKV cases in the department of Tolima ( Table 1).

**Table 1.** ZIKV incidence rates (cases/100,000 pop.) by municipality in the Tolima department and Ibagué communes, Colombia, 2015–2016.*

Municipality*	Cases (2015–2016)	% Cumulated	Population (2016)	Rates (cases/ 100,000 pop.)
Whole department	4,094	100.0	1,412,230	289.9
Carmen de Apicalá Dolores Piedras Flandes Melgar	99 63 44 222 252	2.42 3.96 5.03 10.45 16.61	8,835 8,015 5,640 29,199 36,339	1,120.5 786.0 780.1 760.3 693.5
Purificacion Espinal Icononzo Alvarado Chaparral Ibague Alpujarra	138 343 48 37 183 2,004 13	19.98 28.36 29.53 30.43 34.90 83.85 84.17	29,412 76,149 10,894 88,16 47,248 558,815 4,974	469.2 450.4 440.6 419.7 387.3 358.6 261.4
Lerida Guamo Prado Natagaima Coello Suarez Saldaña Coyaima Rovira Mariquita Falan San Antonio	42 76 18 49 18 8 25 47 34 54 14 21	85.20 87.05 87.49 88.69 89.13 89.33 89.94 91.08 91.91 93.23 93.58 94.09	17,395 32,113 7,701 22,516 9,810 4,547 14,385 28,335 20,542 33,329 9,211 14,310	241.4 236.7 233.7 217.6 183.5 175.9 173.8 165.9 165.5 162.0 152.0 146.8
Valle del San Juan Cunday Ambalema Honda Ataco San Luis Ortega Armero (Guayabal) Libano Venadillo Fresno	6 9 6 21 19 14 18 6 17 7 10	94.24 94.46 94.60 95.11 95.58 95.92 96.36 96.51 96.92 97.09 97.34	6,368 9,634 6,755 24,547 22,589 19,153 32,431 11,839 40,266 19,652 30,165	94.2 93.4 88.8 85.6 84.1 73.1 55.5 50.7 42.2 35.6 33.2
Cajamarca Villahermosa Villarrica Herveo Palocabildo Planadas Rioblanco Anzoategui	5 2 1 1 1 3 2 1	97.46 97.51 97.53 97.56 97.58 97.66 97.70 97.73	19,641 10,652 5,389 8,008 9,160 29,974 24,459 18,638	25.5 18.8 18.6 12.5 10.9 10.0 8.2 5.4
Casabianca Murillo Roncesvalles Santa Isabel Unknown	0 0 0 0 93	97.73 97.73 97.73 97.73 100.00	6,661 5,018 6,344 6,357 -	0.0 0.0 0.0 0.0 -
Ibague commune*	Cases (2015–2016)	% Cumulated	Population (2016)	Rates (cases/ 100,000 pop.)
7	218	10.88	42,370	514.52
9 12 8 4 6 5 11 1 3 13	235 151 270 153 171 99 99 97 70 47	22.60 30.14 43.61 51.25 59.78 64.72 69.66 74.50 77.99 80.34	62,635 42,085 76,141 43,186 48,770 28,902 29,262 30,450 23,426 15,953	375.19 358.79 354.60 354.28 350.63 342.53 338.32 318.56 298.81 294.62
10 2	96 84	85.13 89.32	42,558 40,997	225.57 204.89
Rural area Unknown	14 200	90.02 100.00	32,080 -	43.64 -

*Up to epidemiological week 9 ^th^, March 5, 2016

**Figure 2.**
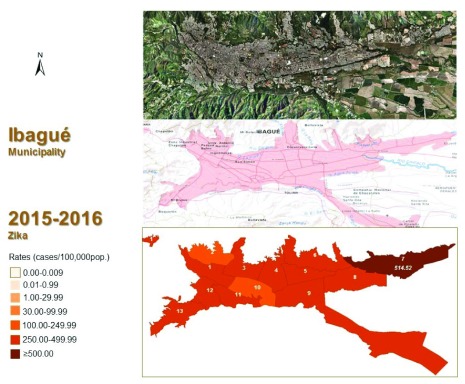
Geographic distribution of ZIKV incidence rates (cases/100,000 pop.) in Ibagué municipality, Colombia, 2015–2016. (*Up to the 9th epidemiological week, March 5, 2016). Aerial photography obtained from the Geographical Institute Agustin Codazzi, Colombia, at: http://ssiglwps.igac.gov.co/ssigl2.0/visor/galeria.req?mapaId=44

For the Ibagué communes, rates ranged from 43.64 (rural area) to 514.52 cases/100,000 pop. (commune 7, 10.88% of the municipality’s cases, located at the east of the municipality) ( Figure 2), followed by commune 9 (375.19 cases/100,000 pop.; 11.73%) and commune 12 (358.79 cases/100,000 pop.; 7.53%). These three communes do not share a common border. The other eight communes had incidence rates ranging between 250–499.99 cases/100,000 pop. ( Table 1, Figure 2). Only three communes had rates higher than the whole Ibagué municipality and of them, only one with a rate >500 cases/100,000 pop. (commune 7) ( Table 1, Figure 2). Five communes (7, 9, 12, 8 and 4) concentrated more than 50% of the cases of the Ibagué municipality and more than 25% of the whole department ( Table 1).

Colombia have officially reported a total of 51,473 cases (up to the 9 ^th^ epidemiological week of 2016); almost 8% from Tolima (4,094). There, burden of ZIKV infection has been concentrated in its east area, were those municipalities with >500 cases/100,000 pop. border two other departments, Cundinamarca (3,778 cases) and Huila (5,338 cases), also with high incidences of ZIKV infection ( Figure 1). Seven municipalities ranged from 250–499.99 cases/100,000 pop., of them five border with high incidence municipalities, including the capital where almost half of the reported cases of ZIKV in Tolima are concentrated ( Figure 1).

## Discussion

Given the ecoepidemiological conditions, particularly of these municipalities, they are now becoming endemic for ZIKV. They have been also endemic of dengue and CHIKV ^7^. Among ZIKV cases in Tolima, 427 (10.43%) were in pregnant women (28 confirmed by RT-PCR for ZIKV) ^6^. Particularly, detailed evaluation of pregnant women morbidity and its mapping due to this arbovirus should be performed ^8, 9^. Even more, the enhanced surveillance of ZIKV-associated neurological syndromes reported eight cases in Tolima as well as three cases of acute flaccid paralysis with history of ZIKV infection ^6^. Public health policies and strategies for integral control of ZIKV in people living, but also in visitors ^10^, in these areas, should be considered and urgently implemented, particularly in the capital, Ibagué. At Ibagué, as well as Tolima, other arboviruses, such as dengue and chikungunya are also cocirculating.

Although ZIKV was isolated in 1947 ^1^, only significant research has been done during the past months (ending 2015-beginning 2016) ^11^, in countries such as Brazil and Colombia in particular, due to multiple negative potentially linked outcomes.

Use of GIS-based epidemiological maps allows for the integration of preventive and control strategies, as well as public health policies, for joint control of this vector-borne disease in this and other areas of the country ^4, 5^. As other arboviruses are cocirculating (dengue, CHIKV and ZIKV), maps for each as well as for coinfections are needed ^12, 13^. Simultaneous or subsequent arboviral infections occur and should be also assessed. Preparedness in this setting should also consider the potential arrival of Mayaro and yellow fever in *Aedes* infested areas. Finally, maps provide relevant information in order to assess the risk of travelers to specific destinations in high transmission areas allowing detailed prevention advice. Migrant and traveler populations also play an important role in the virus spread as they would arrive viremic from endemic areas to non-endemic areas, with vectors that may allow transmission to susceptible individuals ^4, 5, 10^, as occurred in Colombia (including the Tolima department) in 2015–2016.

## Ethics

This study was approved by the Secretary of Health of Tolima IRB as not requiring ethics approval given the study is about secondary grouped data.

## Data availability

The data referenced by this article are under copyright with the following copyright statement: Copyright: © 2016 Rodriguez-Morales AJ et al.

Data associated with the article are available under the terms of the Creative Commons Zero "No rights reserved" data waiver (CC0 1.0 Public domain dedication). 

*F1000Research*: Dataset 1. Raw data for 'Mapping Zika virus infection using geographical information systems in Tolima, Colombia, 2015–2016', 10.5256/f1000research.8436.d118256 ^14^
