# Supplementary material for: Mapping Zika virus infection using geographical information systems in Tolima, Colombia, 2015-2016
Source: F1000Res. 2016 Apr 5;5:568. [Version 1] doi: 10.12688/f1000research.8436.1 (PMC4837980; doi:10.12688/f1000research.8436.1)
Supplement: Peer review history

# Peer review: Referee response for version 1

**DOI:** 10.5256/f1000research.9083.r13330

**Published:** 2016-04-13

**Authors:** Luis Haro-García

**Affiliations:** 1 Department of Public Health, Faculty of Medicine, National Autonomous University of Mexico, Mexico City, Mexico

The manuscript illustrated–through geographic mapping–the epidemiological behavior of Zika virus infection in the municipality of Tolima, Colombia, which results in an easy and understandable way for the decision makers in order to face an emerging problem like the one analyzed.

I think the title and the abstract are accurate; the methodology clearly stands that the study was conducted at one of the high incidence departments located in the central area of ​​Colombia. In general, I think the results are arranged clearly enough; besides, the findings in the municipality of Ibagué, capital of the municipality, given that it comprising almost 40% of the total population of the department, the data are shown independently

It would be desirable at a given moment to develop this same method of mapping conjointly with the municipalities of Cundinamarca and Huila, bordering areas with Tolima, Colombia, where there was also a high incidence of Zika virus infection, at least until March 5, 2016.

The article highlights, in a balanced manner, the advantages of performing this type of mapping, considering that the authors also note the area on study as endemic for dengue and chikungunya.

I have read this submission. I believe that I have an appropriate level of expertise to confirm that it is of an acceptable scientific standard.

# Response

**Published:** 2016-04-14

**Authors:** Alfonso Rodriguez-Morales

**Affiliations:** Universidad Tecnológica de Pereira, Colombia

Thanks for your valuable and positive comments regard this paper. We fully agree with about the assessment of Cundinamarca and Huila Zika incidence rates, given the fact these are bordering areas with Tolima, Colombia, where there was also a high incidence of Zika virus infection. In a future paper we will perform that for those other areas of the country.

# Peer review: Referee response for version 1

**DOI:** 10.5256/f1000research.9083.r13208

**Published:** 2016-04-06

**Authors:** Kateryna Kon

**Affiliations:** 1 Department of Microbiology, Virology and Immunology, Kharkiv National Medical University, Kharkiv, Ukraine

The article provides very interesting information on geographical mapping of Zika virus in Tolima (Colombia). The title and abstract are totally appropriate and represent an adequate summary of the article. There is a comprehensive explanation of the study design with detail description of all methods used, and with appropriate citations. Results are well illustrated in table and figures, and the article is written in grammatically correct and well-understandable scientific language. The conclusions are balanced and totally justified on the basis of the results. All sufficient information has been provided for replication of calculations performed by authors. For the further researches, it would be interesting to compare provided by authors results with results obtained in other areas of Colombia and with results from other countries of Latin America.

I have read this submission. I believe that I have an appropriate level of expertise to confirm that it is of an acceptable scientific standard.

# Response

**Published:** 2016-04-06

**Authors:** Alfonso Rodriguez-Morales

**Affiliations:** Universidad Tecnológica de Pereira, Colombia

Thanks for your comments. We fully agree with all your appreciations. In the near future, when other similar studies would be published we expect to make those comparisons.
